# Collagenous Gastritis a Rare Disorder in Search of a Disease

**DOI:** 10.4021/gr564w

**Published:** 2013-09-09

**Authors:** Rohan Mandaliya, Anthony J. DiMarino, Sheeja Abraham, Ashlie Burkart, Sidney Cohen

**Affiliations:** a Department of Internal Medicine, Abington Memorial Hospital, USA; b Department of Gastroenterology and Hepatology, Thomas Jefferson University Hospital, USA; c Pediatric Gastroenterology, Thomas Jefferson University Hospital, USA; d Department of Pathology, Thomas Jefferson University Hospital, USA; e Department of Gastroenterology and Hepatology, Thomas Jefferson University Hospital

**Keywords:** Collagneous gastritis, Nodular mucosa, Collagenous sprue, Collagenous colitis

## Abstract

A 19-year-old young male presented with abdominal pain and constipation. Subsequent EGD showed nodular gastric mucosa with simple gastric aspirate demonstrating acidic pH of 2.0. The gastric biopsy showed thick subepithelial band of about 15 microns that was confirmed to be collagen on Masson’s trichrome stain along with inflammatory infiltrate. Colonoscopy and capsule endoscopy findings were unremarkable as well as the biopsy of the colon. Collagenous gastritis is a rare histopathological entity characterized by the presence of thick subepithelial collagen band of thickness greater than 10 microns along with intraepithelial lymphocytes and lamina propria lymphoplasmacytic and eosinophilic infitrates. Clinical presentation varies and depends more on the age of the patient with anemia or epigastric pain with nodular gastric mucosa being more common in children while diarrhea being more common in adults due to its increased association with collagenous colitis. The purpose of this case report is; (A) To define the endoscopic and histopathological features and progression of collagenous gastritis in this patient; (B) To compare these findings to those of collagenous sprue and collagenous colitis.

## Introduction

Collagenous gastritis is a rare histopathological disorder of unknown etiology characterized by the deposition of a subepithelial collagen band with accompanying inflammatory infiltrate [1]. It has been reported both in children and in adults. Collagenous gastritis is characterized in children by abdominal pain and anemia due to gastrointestinal bleeding [2-4]. Adult onset collagenous gastritis is frequently associated with collagenous colitis, lymphocytic gastritis, celiac sprue or autoimmune disorders. It may have a wide range of presentations such as watery diarrhea, dyspepsia, weight loss, rectal bleeding or irritable bowel symptoms [1, 2, 5-8]. Most cases have been reported in the pathology literature rather than in clinical gastroenterology journals.

The purpose of this study is threefold: First, to review a single case demonstrating the clinical entity of collagenous gastritis; second, to review the current literature concerning this rare condition; third, to compare collagenous gastritis to two well recognized conditions, collagenous sprue and collagenous colitis, with known clinical sequelae and outcomes.

## Case Report

An 18-year-old male presented with a history of chronic intermittent abdominal pain and constipation for about 6 months. Physical examination was unremarkable. He was thought to have functional constipation and was prescribed Miralax and an increased fiber diet. On follow up visit he did continue to have intermittent abdominal pain and constipation. His basic serological studies were completely normal. Liver function tests were unremarkable. Imaging studies including abdomen x ray and ultrasound were normal. His celiac, thyroid and pancreatic enzyme studies were normal.

**Figure 1:**
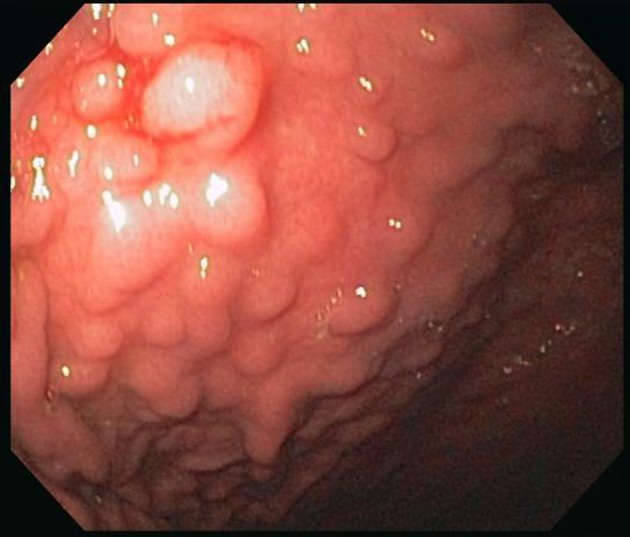
Endoscopy. There is a nodular appearing gastric mucosa prominent in the gastric body and antrum. These nodules are well demarcated.

**Figure 2:**
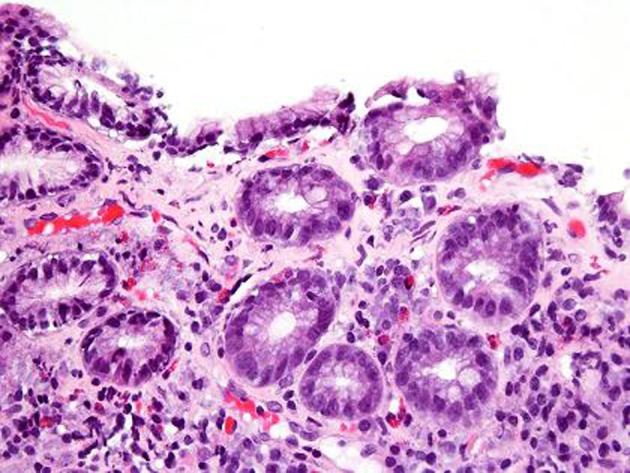
H and E stain. H and E stain shows surface disruption of the inflamed gastric mucosa with intraepithelial lymphocytosis. The lamina propria contains a mixture of plasma cells, lymphocytes and eosinophils. There is capillary trapping and vascular dilatation.

**Figure 3:**
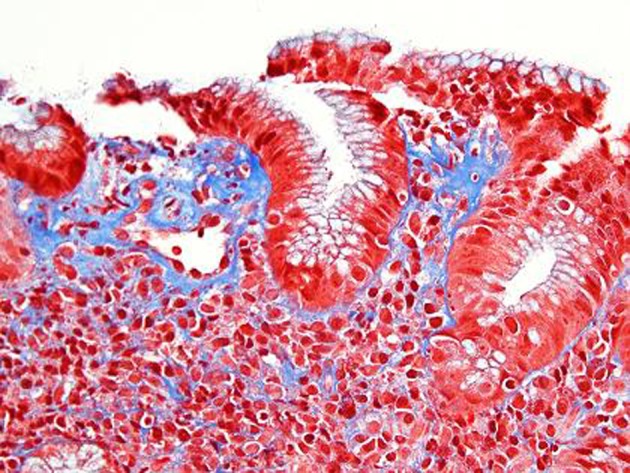
Trichrome stain. Corresponding Trichrome stain shows irregular thickening of the supepithelial band and increased collagen deposition around dilated capillaries. The lamina propria contains a lacelike pattern of increased collagen. In the most affected area the collagen deposition measures 15 microns.

An EGD was subsequently done which showed nodular mucosa of the stomach and peptic duodenitis (Fig. 1). The gastric aspirate showed an acidic pH of 2.0. Gastric mucosal biopsy showed infiltrates on H&E stain (Fig. 2). The trichrome stain showed thickened collagen mucosal band of approximately 15 microns (Fig. 3). Colonoscopy and biopsy were normal. He was diagnosed as collagenous gastritis. He was started on proton pump inhibitors for 3 months. A subsequent autoimmune evaluation including myleperoxidase antibody, proteinase 3 antibody, parietal cell antibody, ANCA and CLO test was negative.

**Figure 4:**
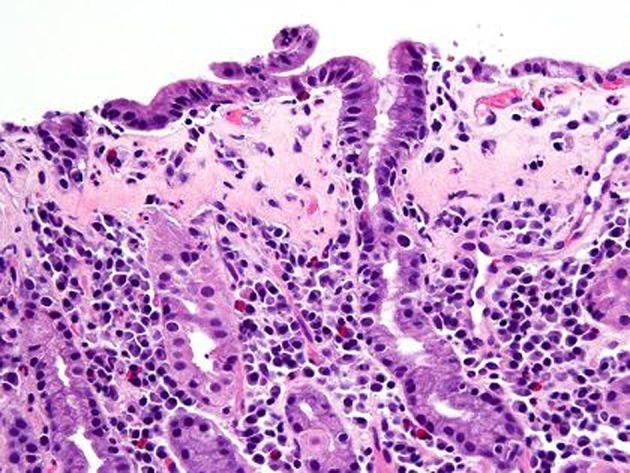
H and E stain after 6 months. Compared to previous findings, follow up gastric biopsy after 6 months shows flattening of the surface foveolar epithelium with increased intraepithelial lymphocytes. There is also lymphoplasmacytic expansion of the lamina propria.

**Figure 5:**
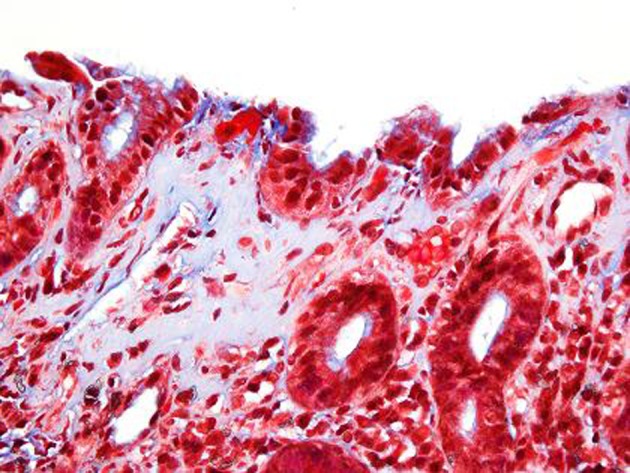
Trichrome stain after 6 months. Corresponding trichrome stain shows prominent subepithelial basement membrane thickening in a well organized pattern with increased collagen deposition measuring up to 25 microns in thickness which was previously 15 microns in thickness.

A repeat endoscopy after 6 months showed persistent finding of a nodular gastric mucosa. Biopsies showed progression of the collagenous gastritis with greater inflammatory infiltrate as well as an increase in the collagen band thickness (Fig. 4, 5). A capsule endoscopy was performed which did not show any abnormality in the small bowel. His constipation was improved on diet therapy and daily Miralax. His abdominal pain decreased in severity.

## Discussion

Collagenous infiltrative inflammation of the gastrointestinal mucosa tract was first described in small intestine as collagenous sprue, then in large intestine as collagenous colitis. More recently it has been described in stomach as collagenous gastritis. The common histologic characteristic lesion in this infiltrative disorder is the presence of subepithelial hyaline like deposit representing collagen. The thickness of the collagen band is usually above 10 microns. It has become recognized recently as a spectrum of new disorders that involve only the columnar mucosa of the gut. It was initially found as an isolated infiltration of a region of the gastrointestinal tract but more recently concomitant involvement of different sites within the gastrointestinal tract has been noted. Collagenous disorders of the gut have been known to cause significant morbidity in middle aged and elderly women with watery diarrhea most frequently [9]. Of the three subgroups, collagenous colitis is the most common. Table 1 compares the three reported collagneous disorders, comparing clinical features and findings.

**Table 1:** Comparison of the Three Collagenous Disorders of the Gut

	Collagenous Colitis	Collagenous Sprue	Collagenous Gastritis
Involvement	Colon	Small Intestine, mainly proximal	Stomach
First Described	1976 by Windstorm	1970 by Winestein	1989 by Colleti and Trainer
Gender Prevelance	Female: Male 20:1	Female > Male 2:1	Not many cases to comment on gender prevelance
Age Prevelance	> 40 years, Peak in 6th and 7th decades	40 - 80 years	Children and Adults
Most common symptoms	Watery non bloody diarrhea	Chronic diarrhea due to malabsorption, Weight loss	Abdominal pain, anemia in children Chronic watery diarrhea, irritable bowel symptoms in adults
Associated Drugs	NSAIDS, SSRI, Ranitidine. Possibly Aspirin, Acarbose, ticlopinine, PPI		
Autoimmune diseases association	Celiac disease, thyroiditis, collagenous gastritis, collagenous colitis	Celiac disease, collagenous gastritis, collagenous colitis	Isolated in most children Adult form associated with collagenous colitis, lymphocytic gastritis, celiac disease and other autoimmune diseases
Colonoscopy findings	Mostly normal appearance to mucosal edema or hyperemia in some	Non specific: loss of mucosal folds, scalloping, mucosal erythema, mosaicism	Normal mucosa, diffuse gastric erythema, erosions, gastric hemorrhages, nodular mucosa
Biopsy findings	Chronic inflammatory infiltrate of plasma cells, lymphocytes and eosinophils in lamina propria with thickened subepithelial collagen bands ( > 10 mm)	Villous atrophy, crypt atrophy, chronic inflammatory infiltrate of plasma cells, lymphocytes and eosinophils in lamina propria with thickened subepithelial collagen bands ( > 10 mm)	Chronic inflammatory infiltrate of plasma cells, lymphocytes and eosinophils in the lamina propria with thickened subepithelia collagen bands (> 10 mm)
Treatment	-Spontaneous remission in some -Loperamide, sulfasalazine, bismuth, cholestyramine. -Budesonide/Corticosteroids -Azathioprine/Methotrexate -Colectomy/ileostomy	-Gluten free diet -Corticosteroids -Azathioprine/Methotrexate -Total parenteral nutrition -Intestinal transplant	-No definite therapy -Dietary modification -The following have been tried with varying results: sucralfate, ranitidine, mesalamine, elimination of gluten, loperamide, cholestyramine, budesonide and prednisione
Complications	Colonic fractures after endoscopic instrumentation, colonic ulceration due to concomitant NSAID use. Evolution into Ulcerative colitis and Crohns disease have been reported	Nutrient deficiencies and progressive weight loss due to malabsorption. T cell Lymphoma similar to as in Celiac disease, Ulcerative jejunitis have been reported	Anemia in children Rare: Upper GI bleed, perforating gastric ulcer, dysphagia due to Plummer Vinson syndrome reported in adults
Clinical Outcome	-Good	-Poor with only few cases reported to have complete resolution of symptoms	-Fair but mostly unknown

Collagenous gastritis is a rare entity. Lagorce-Pages et al describes two subsets of patients: 1), the first type occurs in children and young adults and presents with chronic anaemia due to gastrointestinal bleeding, a nodular pattern on endoscopy, and a disease limited to the gastric mucosa without evidence of colonic involvement; and 2), the second form of collagenous gastritis is associated with collagenous colitis occurring in adult patients presenting with chronic watery diarrhea [2]. Our patient is a young adult who has isolated collagenous gastritis.

The pathogenesis of collagenous gastritis is not known. Because of the similarity and frequent association with collagenous colitis, the same pathogenic mechanisms have been suggested. These mechanisms include chronic inflammation, or autoimmune responses to toxic or infectious agents with proliferation of pericryptal fibroblasts and collagenization of exudated plasma proteins [2, 10, 11]. In support of this theory is the finding that the type of collagen deposited is types I and III, which are known to be associated with inflammation and repair [2]. The question of why collagen deposition is not seen in other form of gastritis including Helicobacter pylori infections, or gastric mucosal injury secondary to non steroidal drugs is not known. Pulimood et al in an ultrastructural study of gastric biopsy specimens from their patient with collagenous gastritis found eosinophil and mast cell degranulation [12]. Mast cells induce migration and proliferation of fibroblasts and stimulate collagen synthesis which may have contributed to the pathogenesis of thickening collagen band. This degranulation could be caused by an unidentified intraluminal antigen, whose continued presence in the diet would explain why this disease is so refractory to pharmacologic treatment. Is there any role of diet in the development of collagenous gastritis, needs to be studied.

Endoscopic findings may show normal mucosa, diffuse gastric erythema, erosions, gastric hemorrhages and nodular mucosa. Mucosal nodularity is one of the characteristic findings of collagenous gastritis seen in children and young adults. A similar nodular appearance may be observed in superficial-type malignant lymphoma of the stomach due to the mucosal involvement of lymphoma cells. These uniform nodules with irregular grooves or erosions have some resemblance to cobblestones. In collagenous gastritis, mucosal nodules are more irregular in size and spread widely to the greater curvature of the gastric body and antrum [13]. The nodular appearance is caused not by swollen mucosa, but depressed mucosa surrounding the nodules. Young patients often show a gastric nodular pattern on endoscopy; in contrast, adult cases show mucosal erythema, erosions to normal mucosa and very rarely nodular mucosa as seen in one report [14]. It is not clear why the adults do not show mucosal nodularity although the histological findings are similar to those in young adults and children.

Gastric pH has been studied in patients with collagenous gastritis. In our patient simple gastric aspirate was acidic at a pH of 2.0. No quantitative measure was done. Winslow et al performed a continuous 24-hour gastric pH monitoring. It demonstrated a pH less than 2 and appropriate inhibition by ranitidine (namely, pH > 4, 45% of the time) [15]. It implies that patients with collagenous gastritis do not have achlorhydria as seen in atrophic gastritis. However at the same time we cannot attribute gastric changes and collagen deposition to gastric acid output.

The diagnosis of collagenous gastritis is based on the histological findings on gastric biopsies which reveal thick collagenous bands within the subepithelium of the gastric mucosa along with an inflammatory infiltrate. It is identified with trichrome stain. Viseoulis et al in their pathological findings compared the collagen band thickness in 10 controls with normal gastric mucosa and 10 patients with chronic gastritis; the mean collagen band thickness was 1.37 and 1.19 microns, respectively. In contrast, antral biopsies in patient with collagenous gastritis showed mean thickness of subepithelial collagen of 27.07 microns [7]. The range of collagen band thickness in reported cases has been between 10 - 120 microns. A clear criterion for the thickness of the collagen band is not defined but it has been hypothesized that the findings are similar to previously defined collagenous colitis with collagen thickness of greater than 10 microns. The inflammatory cell response is predominantly a mononuclear infiltrate with few neutrophils and eosinophils in the lamina propria. The number of intraepithelial lymphocytes often exceeds 20 per 100 epithelial cells (normal 3 - 5 per 100 epithelial cells) [2]. Thus the histologic features of collagenous gastritis include lamina propria lymphoplasmacytosis with eosinophils, patchy surface lymphocytosis, patchy subepithelial collagen deposition of variable thickness as demonstrated by Masson’s trichrome stain, injury and detachment of surface epithelium, and glandular atrophy.

No definite treatment has been proven in this condition. Various treatment options have been tried with varying results. They include prednisone, budesonide, sucralfate, ranitidine, mesalamine, elimination of gluten, loperamide and cholestyramine. Dietary antigen may play a role in the development of the collagen band and identification of the antigen may lead to effective dietary treatment similar to a gluten free diet in celiac disease, Pulimood et al [12].

Prospective studies in patients with collagenous gastritis have not been reported with only case reports being published. The natural history of collagenous gastritis with or without colitis is not well documented. In adults, a chronic intermittent course characterizes the majority of patients, with no significant mortality risk or severe progression [16]. Diarrhea may resolve with or without treatment, although relapses may occur. In children, a more recalcitrant disease is suspected. A few reports have documented that the abnormal collagen band persists despite symptomatic relief in patients with or without medication [2, 4, 11].

Winslow et al describes the clinicopathologic evolution of collagenous gastritis in a single patient during a 12-year period [15]. A hundred and nine gastric biopsy specimens from 19 different endoscopic procedures were evaluated for severity and distribution of collagenous gastritis in this patient. Relative to biopsy specimens from age and sex matched control subjects, the patient’s biopsy specimens showed a significantly lower number of antral gastrin cells, along with a significant corpus endocrine cell hyperplasia, suggesting an increased risk of endocrine neoplasia.

The present case study illustrates the current problem in better defining this disorder. A clear cut pathological entity similar if not identical to the well defined clinical entities of collagenous sprue and collagenous colitis, lacks clinical definition, etiology and prognosis. The etiology and treatment options need to be studied further. The condition is possibly under diagnosed at this point of time and the presence of collagenous gastritis should entail further workup for the presence of associated collagenous colitis, collagenous sprue or celiac disease.
